# Anti-Metastatic Function of Extracellular Vesicles Derived from *Nanog*-Overexpressing Melanoma

**DOI:** 10.3390/curroncol29020088

**Published:** 2022-02-11

**Authors:** Tomohiro Hatakenaka, Nahoko Matsuki, Seiya Minagawa, Celine Swee May Khoo, Mikako Saito

**Affiliations:** 1 Department of Biotechnology and Life Science, Tokyo University of Agriculture and Technology, 2-24-16, Naka-cho, Koganei, Tokyo 184-8588, Japan; hatakenaka.tomohiro@gmail.com (T.H.); s208361s@st.go.tuat.ac.jp (N.M.); s212366u@st.go.tuat.ac.jp (C.S.M.K.); 2 Department of Industrial Technology and Innovation, Tokyo University of Agriculture and Technology, 2-24-16, Naka-cho, Koganei, Tokyo 184-8588, Japan; s205244t@st.go.tuat.ac.jp; 3 Bioresource Laboratories, Tokyo University of Agriculture and Technology, 2-24-16, Naka-cho, Koganei, Tokyo 184-8588, Japan

**Keywords:** melanoma, *Nanog* overexpression, extracellular vesicles, autovaccine, TGF-β1

## Abstract

A metastatic melanoma cell line B16-F10 (F10) was modified to a more undifferentiated state by *Nanog* overexpression. The produced cell line *Nanog*^+^F10 showed a higher metastatic potential than F10. Instead of whole cells, the extracellular vesicles (EVs) therefrom were investigated about their possible role as an autovaccine against metastasis. EVs from *Nanog*^+^F10 cells (*Nanog*^+^F10-EVs) could suppress the metastasis, contrasting the EVs from less metastatic F10 cells (F10-EVs) enhanced metastasis. The involvement of TGF-β1 in the role of *Nanog*^+^F10-EVs was analyzed, as TGF-β1 was a secretory cytokine being affected most intensively by *Nanog* overexpression. It was suggested to be crucial that the TGF-β1 concentration in *Nanog*^+^F10-EVs should be as low as 1.6 pg/μg for its metastasis-suppressive role. In response to *Nanog*^+^F10-EVs, immunoreaction was observed in liver, indicating the specific decrease in the number of tumor-promotive CD163-positive macrophages. These indicate a possibility of *Nanog*^+^F10-EVs as a novel autovaccine candidate against melanoma metastasis.

## 1. Introduction

Extracellular vesicles (EVs) are nano-sized vesicles released by various cell types that have functions of cell-to-cell communication at various distances, maintenance of organ homeostasis, and induction of diseases [1,2,3]. It is expected that the functions of EVs will reflect the properties of the original cells that released those EVs.

A nutritive microenvironment or niche is required for disseminating tumor cells to engraft distant sites. The concept of a niche is based on the ‘seed and soil’ hypothesis [4]. Pancreatic ductal adenocarcinoma-derived EVs, for instance, may induce formation of a pre-metastatic niche in the liver [5]. Metastatic niche formation is not limited to distant sites. Prostate cancer-associated EVs modulate the tumor microenvironment to prepare a metastatic niche [6]. EVs containing ErbB2/CRK derived from bladder cancer can induce pre-metastatic niches in lung, liver, and bone [7]. Tumor cell-derived EVs are believed to stimulate tumorigenesis via promoting angiogenesis [8], remodeling of extracellular matrix [9], secretion of inflammatory molecules, and suppression of immune responses [10,11].

By contrast to EVs from tumor cells, EVs of histiocytes, immune cells, or cancer cells with low metastatic potential may suppress cancer metastasis; this latter property may be of value for anti-metastasis therapies using an EV-delivery system. Intravenous administration of EVs isolated from non-metastatic melanoma cells has been shown to suppress the metastasis of melanomas [12]. Similarly non-cancer cell-derived exosomes, whose stability in blood has been increased by the expression of CD47, are effective in treatments targeting the Kras oncogene in pancreatic cancer [13]. EVs of dendritic cells can promote a tumor antigen-specific response [14]. Menstrual mesenchymal stem cells inhibit angiogenesis and the growth of oral squamous cell carcinomas [15]. MC38, a colorectal adenocarcinoma model cell line, has been genetically modified by overexpressing IL-12 to enhance its natural killer cell activation potency; EVs from this modified MC38 cell line can suppress the growth of subcutaneous tumors in the abdominal cavities of mice [16].

However, given the diversity of cancer cells and the complex factors associated with their metastasis, we posited that the characteristics of EVs might not always reflect the original cancer cells. Moreover, we wondered if highly aggressive cancer cell-derived EVs could be modified into suppressive EVs by cytokines and immune cells during in vivo circulation. If EVs with such properties are available, their role may be an autovaccine against metastasis [3].

Cancer vaccines have been intensively studied and properties of tumor antigens are summarized in a recent review [17]. Vaccine types are mostly peptide, glycopeptide, viral, DNA, and mRNA. Although the number is small, there are also cellular type such as dendritic cells, melanoma cells, colon cancer cells, and pancreatic adenocarcinoma cells. However, there is no report on subcellular particles and extracellular vesicles. It seemed to be due to an idea that those fractions might not contain cancer antigens that should exist on the cell surface. In contrast, our idea is that EVs may provide intracellular molecular information to the immune system of the target tissue rather than cell surface antigen information. Then, instead of whole cell, EVs have been investigated on their possible role as a candidate of autovaccine.

Previously, we modified the melanoma cell line B16-BL6 (BL6) to overexpress *Nanog*, a major factor for maintaining the undifferentiated state of embryonic stem cells, in the expectation that this would increase the metastatic potential of this cell line. The modified BL6 line did indeed show an enhanced level of metastatic potential [18]. A transcriptome sequencing analysis indicated that transforming growth factor (TGF)-β1 was the only secretory cytokine among the top15 up-regulated and top 16 down-regulated genes after *Nanog* overexpression in BL6 cells.

Here, we have modified the melanoma cell line F10 to overexpress *Nanog*. F10 and BL6 are the same strain of melanoma, but with completely different metastatic properties. BL6 does not metastasize to the liver, but F10 most often metastasizes to the liver. Therefore, regarding the effects of *Nanog* overexpression, although the effect on BL6 was investigated in the previous report, it is necessary to confirm that the same effect on F10 could be obtained as well.

The involvement of TGF-β1 was also assumed in the effects brought about by *Nanog* overexpression. TGF-β1 shows a dual role in the regulation of metastasis; tumor-suppressive in early stage tumors, but tumor-promotive in advanced cancer [19,20,21]. Moreover, TGF-β1 can be transferred to distant target cells through EVs [22]. Therefore, we thought that a key role of TGF-β1 in the suppression of metastasis might be found.

Next, we considered the interaction between TGF-β1 and immune cells. Among immune cells such as macrophages, T-cells, B-cells, and natural killer cells, we focused on macrophages based on the report that TGF-β1 could induce tumor promotive M2-like macrophages [23]. Immune responses concerning M1- or M2-polarization of macrophages might be related to the metastasis suppression via TGF-β1 signaling.

## 2. Materials and Methods

### 2.1. Cell Culture

A mouse melanoma cell line, B16-F10 (F10) cells, and a mouse macrophage cell line, J774.1 cells were cultured in R10 medium (RPMI 1640 containing 10% fetal bovine serum [Thermo Fisher Scientific, Waltham, MA, USA] and penicillin-streptomycin [Thermo Fisher Scientific]) at 37 °C under 5% CO_2_.

### 2.2. Animals

This study was carried out in compliance with the ARRIVE guidelines [24]. C57BL/6 male mice were bred in a specific pathogen-free room under conditions of 12 h illumination and 12 h darkness each day. Every mouse was fed a solid diet (MF, Oriental Yeast Co., Ltd., Tokyo, Japan) and 8–9 week old mice were used in the experiment. Animal experiments were conducted in accordance with the guidelines of the “Guide for the Care and Use of the Laboratory Animals” of Tokyo University of Agriculture and Technology and were approved by the Institutional Animal Care and Use Committee of Tokyo University of Agriculture and Technology (IACUC No. 30-128 and No. R02-130).

### 2.3. Preparation of a Nanog Overexpressing Cell Line and a TGF-β1 Knockdown Cell Line

An overexpression vector for *Nanog* was constructed by inserting the *Nanog* gene into a pCAG-IRES-PuroR-EGFP. The vector product (4 μg/250 μL RPMI) and a Lipofectamine 2000 solution (5 μL/250 μL RPMI) were mixed and incubated at 25 °C for 20 min. The mixture was added to 90% confluent F10 cells and incubated for 3 h. The cells were then cultured in fresh R10 at 37 °C for 48 h. After replacing the medium with R10 containing 1.5 μg/mL puromycin, cells were cultured for 2 weeks to select the *Nanog* overexpressing cells (*Nanog*^+^F10). A knockdown vector for *TGF-β1* was prepared by inserting the sh*TGF-β1* into pU6-PGK-PuroR. Using this vector, a *TGF-β1* knockdown cell line was prepared in the same protocol as that for a *Nanog* overexpressing cell line. The overexpression of *Nanog* was confirmed by quantitative RT-PCR and western analysis as described below. The knockdown of *TGF-β1* was confirmed by quantitative RT-PCR, western analysis, and ELISA as described below.

### 2.4. Quantitative RT-PCR

Total RNA was prepared by ISOGEN II (Nippongene, Tokyo, Japan) according to the manufacturer’s instructions. The expression levels of *Nanog, EGFP*, *puroR*, *TGF-β1*, *F4/80*, *CD68*, *CD80*, *CD86*, *CD163*, *and CD206* mRNAs were determined by quantitative RT-PCR using StepOnePlus^TM^ Real-Time PCR System (Applied Biosystems, Waltham, MA, USA). The analysis was performed under the following conditions: 95 °C for 10 min, 45 cycles of a reaction set (95 °C denaturation for 15 s, 60 °C annealing for 1.0 min), and a reaction set for melt curve analysis (95 °C for 15 s and 60 °C for 1 min). Primer sets of respective target RNAs are listed in Table S1. The amount of target mRNA was normalized to the amount of *Gapdh* mRNA.

### 2.5. Western Analysis

A protein sample of cells was prepared according to the following procedure. After rinsing 70–80% confluent cells with PBS, an RIPA buffer (25 mM Tris-HCl, 150 mM NaCl, 1% NP-40, 1% sodium deoxycholate, 0.1% SDS, pH 7.6; Thermo Fisher Scientific) was added to the culture dish. The dish was stood on ice for 15 min. Then, the cells were peeled from the culture dish with a cell scraper and collected in a 1.5 mL microtube. The cell suspension was sonicated (UR-20P; TOMY SEIKO, Tokyo, Japan) on ice and centrifugated at 20,000× *g* for 15 min. The supernatant was collected as a protein sample of cells. A protein solution of EVs was prepared as follows. The pellet of EVs obtained as the precipitate of ultracentrifugation was suspended in an RIPA buffer and stood on ice for 15 min. The protein concentration was determined using a Pierce^®^ BCA ™ Protein Assay kit (Thermo Fisher Scientific). A protein solution was mixed with a 1/6 volume of 0.375 M Tris-HCl (pH 6.8) buffer solution containing 93 μg/mL DTT, 0.12 g/mL SDS, 0.6 mL/mL glycerol, and 0.6 mL/mL bromophenol blue. Then, the solution was heated at 95 °C for 5 min and applied to SDS-PAGE at 150 V.

Blotting onto a PVDF membrane was conducted at 100 V for 3 h at 4 °C. The PVDF membrane was then immersed in a TBS-T (Tris-buffered saline (25 mM Tris, pH 7.4, 150 mM NaCl) containing 1 (*v*/*v*) % Tween 20) solution containing 5 (*w*/*v*) % skim milk at 25 °C for 30 min. Then, the PVDF membrane was incubated in a 5% skim milk TBS-T solution containing primary antibody at 25 °C for 3 h. Primary antibodies against mouse Gapdh (1:1000, sc-32233; Santa Cruz Biotechnology, Dallas, TX, USA), mouse Nanog (1:500, ab80892, Abcam, Cambridge, UK), and mouse HSC70 (1:500, sc-7298, Santa Cruz) were used, respectively. After washing with TBS-T three times, the membrane was incubated in TBS-T containing a secondary antibody (anti-mouse immunoglobulin conjugated to alkaline phosphatase, Promega, Madison, WI, USA) at 25 °C for 1 h. Membranes were washed 3 times with TBS-T and then incubated with Western Blue Stabilized Substrate (Promega) for alkaline phosphatase at 25 °C for 5 min. Colored bands of target proteins were quantified using ImageJ software (NIH: https://imagej.nih.gov/ij/ (Access on 30 October 2017).

### 2.6. ELISA

The amount of TGF-β1 in EVs and in culture medium were measured using TGF-beta 1 Quantikine ELISA kit (R&D Systems, Minneapolis, MN, USA) according to the manufacturer’s instructions. After collecting the culture medium from the culture dish, the cells were collected by trypsin/EDTA treatment to estimate the number of cells. The amount of TGF-β1 in the culture medium was divided by the number of cells to determine the amount of TGF-β1 released per cell.

### 2.7. Measurement of Cell Proliferation

F10 and *Nanog*^+^F10 cells were cultured in R10 medium, respectively, in dishes with 6 cm in diameter. The number of starting cells was 1 × 10^5^ cells per dish with 6 cm in diameter. Three dishes were used for the cell count. In total, nine dishes were used for the cell count at 0 h, 48 h, and 96 h. For the dishes for 96 h culture, the medium was replaced at 48 h by fresh R10 for the culture for another 48 h. At respective time points, the cells were harvested by washing with 1 mL PBS twice, treatment with 200 μL trypsin/EDTA for 5 min, adding 2 mL culture medium, and centrifuged at 1500 rpm × 5 min. The precipitate was suspended in 2 mL culture medium and cell count was performed by 8-fold dilutions and with a hemocytometer.

### 2.8. Wound Healing Assay

Test cells were cultured on a 12-well plate until they became confluent. The monolayer cell sheet on the bottom plate in a well was scratched with a sterile plastic chip to form a model of wound. During culture, the microscopic image of this wound area was recorded from 0 h up to 24 h, and the changes of wound area were analyzed using ImageJ software to determine the wound healing rate.

### 2.9. Isolation of EVs

In this study, HSC70 was used as a marker of melanoma-derived exosomes since it was used in common in both references [25,26]. HSC70 was also used as a checking marker of exosome contamination in fetal bovine serum (FBS). FBS was centrifuged at 100,000× *g* for 80 min twice to prepare EV-depleted FBS. A trace of contamination of EVs, if any, might interfere with the EVs collected as the HSC70 positive fraction.

The protocol for the separation of EVs was determined by referring to [27]. Cells were cultured for 48 h and rinsed with PBS. Then, RPMI 1640 medium containing 10% EV-depleted FBS and 1% penicillin streptomycin was added to the culture dish. After the culture for 48 h, the culture medium was collected by centrifugation at 2000× *g* for 20 min. The supernatant was centrifugated at 10,000× *g* for 40 min and then at 100,000× *g* for 80 min. The resulting precipitate was suspended in PBS and passed through a filter with a pore size of 0.22 μm. The filtrate was centrifugated again at 100,000× *g* for 80 min, and the final precipitate was suspended in PBS to obtain a suspension of EVs.

### 2.10. Analysis of Size Distribution of EVs

The size distribution of EVs was analyzed using a NanoSight NS300 system (Malvern Panalytical, WR14 1XZ, Malvern, UK). A 100 μL PBS containing more than 10^7^ particles was prepared and analyzed. The mode diameter and mean diameter were determined by five measurements.

### 2.11. Observation of EVs with an Electron Microscope

The same amount of 4% paraformaldehyde (PFA) and a PBS suspension of EVs were mixed and incubated at 25 °C for 30 min for fixation of EVs. Three μL aliquots of the fixed suspension of EVs were placed dropwise on a grid with a support membrane (JEOL, Tokyo, Japan) that was made hydrophilic and dried at room temperature in advance. The grid was washed with PBS 7 times, fixed with 1% glutaraldehyde for 5 min, washed with pure water 7 times, stained with a 3% phosphotungstate solution for 10 min, and dried at room temperature for 10 min. The sample of EVs on the grid was observed with a transmission electron microscope.

### 2.12. Count of Metastatic Colonies and Estimation of Volume

Melanoma cells (2.5 × 10^5^ cells/250 μL PBS) were injected into the tail vein of 8–9 week old C57BL/6 male mice. Two weeks later, mice were euthanized by cervical dislocation, and the livers were separated into lobes and photographed under a microscope. EVs (5 μg/100 μL PBS) were injected into the tail vein of 5–6 week old mice three times per week for 3 weeks, and subsequently melanoma cells (2.5 × 10^5^ cells/250 μL PBS) were injected into the tail vein. Ellipsoidal major and minor diameters of each metastatic colony were analyzed using ImageJ software, and the volume was calculated by using the following formula; V = 1/6πab^2^, where, a and b are the major and minor diameters, respectively.

### 2.13. Macrophage Depletion

Clodronate was used to deplete the function of macrophages. Clodronate-encapsulated liposomes (12.5 mg/kg mouse weight) (Hygieia Bioscience, Osaka, Japan) were repeatedly injected into the tail vein of mice for 3 weeks at an interval of 4 or 5 d. The effect of clodronate was confirmed by the decrease or loss of the gene expression of 6 macrophage markers and histochemical analysis by hematoxylin-eosin (HE) staining.

### 2.14. HE Staining

A liver of the mouse was embedded in Neg-50 (Thermo Fisher Scientific) and allowed to stand at 4 °C for 18 h. Frozen blocks were prepared using isopentane (FUJIFILM Wako Pure Chemical, Osaka, Japan) immersed in liquid nitrogen. The frozen block was sliced into 7 μm thick tissue sections using a cryostat HM550OVP (Thermo Scientific Microm) and a MAS-coated slide glass (MATSUNAMI, Kishiwada, Osaka, Japan) was pressed against the tissue section for its attachment on the glass. The frozen tissue section on the glass was washed with water and immersed in Meyer-hematoxylin solution (FUJIFILM Wako Pure Chemical) for 10 min for nuclear staining. Subsequently, it was washed with running water for 5 min and immersed in an eosin solution for 2 min to stain the cytoplasm. It was rinsed with water for about 5 s to wash off excess eosin solution, and soaked in 70 (*v*/*v*) % ethanol for 5 min, then in each of 90, 95, and 100 (*v*/*v*) % ethanol for 2 min in this order. After that, it was soaked in xylene for 5 min twice, and encapsulated with a specimen-encapsulating agent New MX (MATSUNAMI), a xylene-based anti-fading agent.

### 2.15. Fluorescent Immunostaining of CD68

Frozen tissue sections were fixed by dropping 4% PFA and allowing to stand for 15 min. It was then washed by immersing it in 10 mM glycine/PBS three times for 5 min. A 2% gelatin solution was added dropwise to the tissue section and allowed to stand for 20 min for blocking. The washing was repeated 3 times by immersing in 10 mM glycine/PBS for 5 min. After further immersing in 0.1% BSA/PBS for 5 min, 1% BSA/PBS containing an antibody against CD68 (1:50, sc-20060, Santa Cruz) was added dropwise, and the mixture was allowed to stand for 40 min. The washing was repeated 5 times by immersing in 0.1% BSA/PBS for 5 min. Next, 1% BSA/PBS containing goat anti-mouse IgG H&L labelled with Alexa Fluor 488 (Ex: 496 nm, Em: 519 nm) (1:200, ab150113, Abcam) was added dropwise and allowed to stand for 40 min. The washing was repeated 5 times by immersing in 0.1% BSA/PBS for 5 min. Counterstaining was performed by immersing in a hematoxylin solution for 30 s and rinsing with a running water for 5 min. Moisture around the section was wiped off with a Kimwipe, dried for about 1 min, then the encapsulant New MX was added dropwise, and the mixture was sealed with a cover glass. The sample was observed with an inverted confocal microscope.

### 2.16. Fluorescence Analysis of the Uptake of EVs by J774.1 Cells

EVs were labelled with SYTO RNASelect™ Green Fluorescent cell Stain (SYTO) (Thermo Fisher Scientific) (Ex: 490 nm, Em: 530 nm) by incubation at 37 °C for 20 min in the dark. A mouse macrophage cell line, J774.1 cells were seeded in a 6-well plate at 2 × 10^5^ cells/well in 2 mL of R10 medium. The next day, cells were washed twice with PBS and 2 mL of EVs-depleted medium was added to the well. Then, 60 μg of fluorescently labeled EVs (F10-EVs-SYTO or *Nanog*^+^F10-EVs-SYTO), SYTO (EVs-less control), or PBS (no treatment control) were added to the well. After incubation at 37 °C for 1 h in the dark, the cells were washed twice with PBS and covered with 2 mL of EVs-depleted medium for the fluorescence image analysis with a confocal microscope. A rectangular area was arbitrarily selected from a microscopic image. The total area of cells (S_cell_) was determined from the bright field image. The total fluorescent intensity (F_cell_) was determined by integrating the fluorescent intensities over S_cell_. The fluorescent intensity per unit area for this image was determined by F_cell_/S_cell_. Five images were analyzed for each of four conditions (F10-EVs-SYTO, *Nanog*^+^F10-EVs-SYTO, SYTO, PBS). The F_cell_/S_cell_ value for PBS condition was subtracted from those for the other conditions.

### 2.17. Fluorescent Immunostaining of CD163 in J774.1 Cells

J774.1 cells were cultured at 2.5 × 10^5^ cells/dish with 20 μg of F10-EVs, *Nanog*^+^F10-EVs, or PBS (control) for 48 h. The cells were collected by trypsin/EDTA treatment and suspended in PBS. An aliquot of cell suspension containing 1.0 × 10^6^ cell was collected and washed twice with PBS containing 3% BSA. Then, the cells were reacted with anti-CD163 antibody (1:10, sc-58965, Santa Cruz) at room temperature for 1 h and subsequently with goat anti-mouse IgG (H + L) Alexa Fluor 568 (1:285.7, A-11004, Invitrogen, Waltham, MA, USA) at room temperature for 30 min. The cells were plated on a glass dish and the fluorescent images were analyzed using ImageJ. The fluorescent intensity and area of every fluorescent cell was integrated for an image to determine the fluorescent intensity per unit area. Eight or nine images were analyzed for every condition and the results of F10-EVs and *Nanog*^+^F10-EVs were expressed as relative values to that of PBS.

### 2.18. Statistics

Specific details regarding statistical analyses are presented in the figure legends. Preparation of test samples for mRNA or protein was done using one test sample per dish. Each test sample was analyzed twice or three times and the average of the two or three results was recorded as the value for one test sample. Results are presented as mean ± standard deviation (SD) or mean ± standard error of mean (SEM) for the number of samples (*n*). Results of metastatic colony analyses are presented in box plots. Outliers shown in box plots were determined by a Smirnoff–Grubbs test to be greater than 0.05 one-tailed probability. The statistical significance between two specific data groups was analyzed by two-tailed Student’s *t* test. The statistical significance of results is denoted by a *p* value or by marking with asterisk(s): ***: *p* < 0.001, **: *p* < 0.01, *: *p* < 0.05, †: *p* < 0.1.

## 3. Results

### 3.1. Metastatic Potential of Nanog Overexpressing F10 Cells

**Figure 1:**
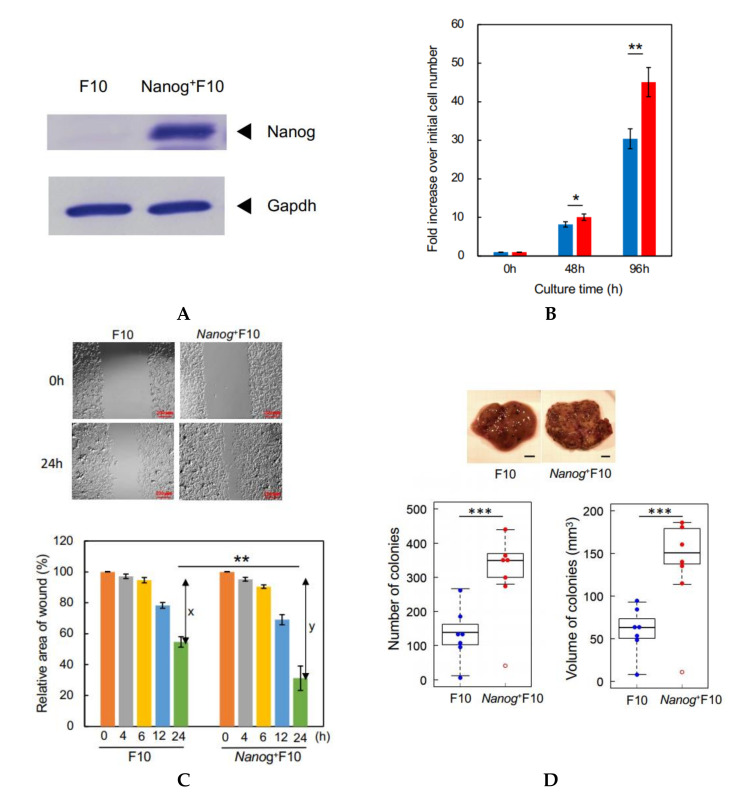
Properties of a *Nanog*^+^F10 cell line. (**A**) Confirmation of Nanog expression in *Nanog*^+^F10 cell. (**B**) Relative rate of cell proliferation in cultures of F10 and *Nanog*^+^F10 cells. mean ± SD, for *n* = 3. (**C**) Wound-healing assay to compare migratory activity of F10 and *Nanog*^+^F10 cells. The smaller the value in the graph, the higher the migratory activity. x and y indicate the healed areas measured at 24 h in respective cultures. Here, x and y were 42% and 69%, respectively. Therefore, y/x was 1.64. Bars are mean ± SD, for *n* = 4. (**D**) Representative images, number, and volume of metastasis colonies in the liver of mice. Scale bar, 5 mm; *n* = 7 excluding outliers (◦). The image of (**A**) was cropped from red dotted lines on full-length gel images shown in Supplementary Figure S1. ***: *p* < 0.001, **: *p* < 0.01, *: *p* < 0.05.

We performed a western analysis to confirm the absence of Nanog in wild-type F10 cells and the presence of Nanog in *Nanog*^+^F10 cells (Figure 1A).

In vitro tests to evaluate metastatic properties are described in [28]. Among them, cell proliferation rate and wound-healing activity were tested in this study. High rate of cell proliferation facilitates the growth of cancer cells at the metastasized sites and may promote the metastasis. High level of the wound-healing activity facilitates the migration of cancer cells and may promote the metastasis.

The rate of cell proliferation of *Nanog*^+^F10 cells was higher than F10 cells: after 96 h, the *Nanog*^+^F10 cells had increased by 45.1 fold, the F10 cells by 30.4 fold (Figure 1B). Wound-healing activity was also enhanced by *Nanog* overexpression. The ability of *Nanog*^+^F10 cells to heal a wound in culture was 1.64 fold higher than F10 cells that was given by y/x (Figure 1C). An in vivo test using mice demonstrated that black metastatic colonies (Figure 1D, photographs) appeared mainly in the liver and lungs. The number of colonies in the liver was greater than that in the lung (Figure S2). Some metastatic colonies were also observed in the kidney and fat of some animals, but the total number of these colonies per mouse was no greater than 2. Therefore, the metastasis to the liver has been focused in this study. The colony volume (Figure S3) was determined by the formula for a spheroid. The smallest colony was about 90 μm in size and 0.0004 mm^3^ in volume. The colony size was distributed from 0.1 to 1.0 mm. *Nanog* overexpression caused a large increase in the liver. The number and volume of colonies in *Nanog*^+^F10 were 2.5 fold and 2.4 fold, respectively, greater than in F10 (Figure 1D).

### 3.2. Metastasis-Suppressive Effect of Nanog^+^F10-EVs

**Figure 2:**
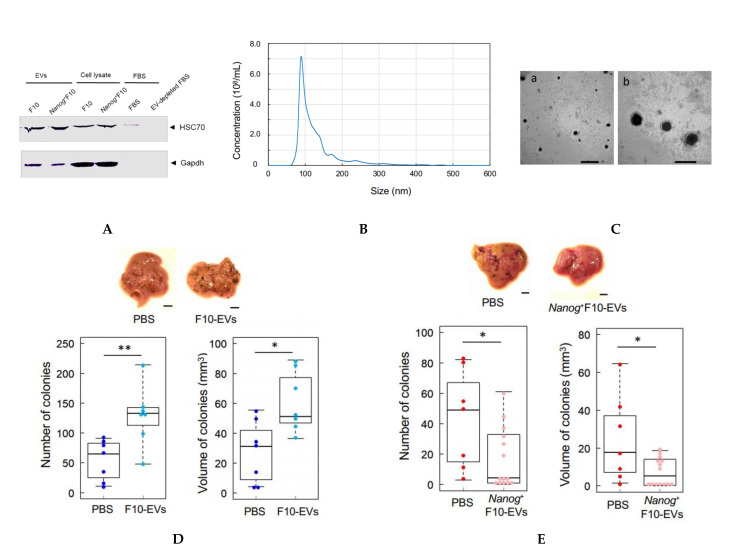
Comparison of the effects of pre-administration of F10-EVs and *Nanog*^+^F10-EVs in mice on the metastasis. (**A**) Western analysis of HSC70 protein in EVs and EV-depleted FBS. The lane of EV-depleted FBS is a sample of FBS after ultracentrifugation at 100,000 g × 80 min twice. (**B**) Particle size distribution of EVs determined by NanoSight analysis. Concentration: 3.07 × 10^10^ ± 0.06 particles/mL, Mode diameter: 90.0 ± 0.7 nm, mean diameter: 130.1 ± 1.0 nm, *n* = 5. (**C**) TEM photographs of EVs. Scale bars: (a) 500 nm, (b) 200 nm. (**D**) Effects of pre-administration of F10-EVs and PBS on the images, number, and volume of metastatic colonies after injection of F10 cells. Scale bar, 5 mm; *n* = 7. (**E**) Effects of pre-administration of *Nanog*^+^F10-EVs and PBS on the images, number, and volume of metastatic colonies after injection of *Nanog*^+^F10 cells. Scale bar, 5 mm; *n* = 14 for *Nanog*^+^F10-EVs, *n* = 7 for the control (PBS). The image of (A) was cropped from red dotted lines on full-length gel image shown in Supplementary Figure S4. **: *p* < 0.01, *: *p* < 0.05.

FBS should not contain EVs brought from outside because culture medium containing FBS is used for the separation of EVs from only melanoma cells. Commercially available FBS, however, may inevitably contain EVs from original animal. The EV-depleted FBS could be successfully prepared (Figure 2A). Using the EV-depleted FBS, melanoma-derived EVs were collected (Figure 2A) and their size distribution was analyzed. The mode diameter and mean diameter were 90 nm and 130 nm, respectively (Figure 2B). TEM analysis revealed that there were many particles with a diameter of ca 100 nm (Figure 2C). EVs prepared in this study were principally exosomes though they contained a small amount of particles greater than 130 nm. According to [29], there are occasions that exosome size ranges 30–250 nm. Particles with a size over 200 nm are difficult to be removed even by ultracentrifugation and filtration. In fact, our EV samples contained such large particles.

An EV suspension (100 μL PBS) containing 5 μg EVs derived from F10 (F10-EVs) was injected into the tail vein of mice three times per week for 3 weeks. Then, F10 cells (2.5 × 10^5^ cells/250 μL PBS) were injected into the tail vein and the mice were maintained for 2 weeks. The number and volume of metastatic colonies in the liver increased 2.0 fold and 1.6 fold, respectively, as compared with controls that received PBS (Figure 2D). In contrast, when mice were injected with EVs derived from *Nanog*^+^F10 (*Nanog*^+^F10-EVs) followed by the injection of *Nanog*^+^F10 cells, the total number and volume of metastatic colonies of *Nanog*^+^F10 cells decreased 0.10 fold and 0.30 fold, respectively, as compared with controls (Figure 2E).

### 3.3. Effects of TGF-β1 Knockdown on the Metastasis Suppression

**Figure 3:**
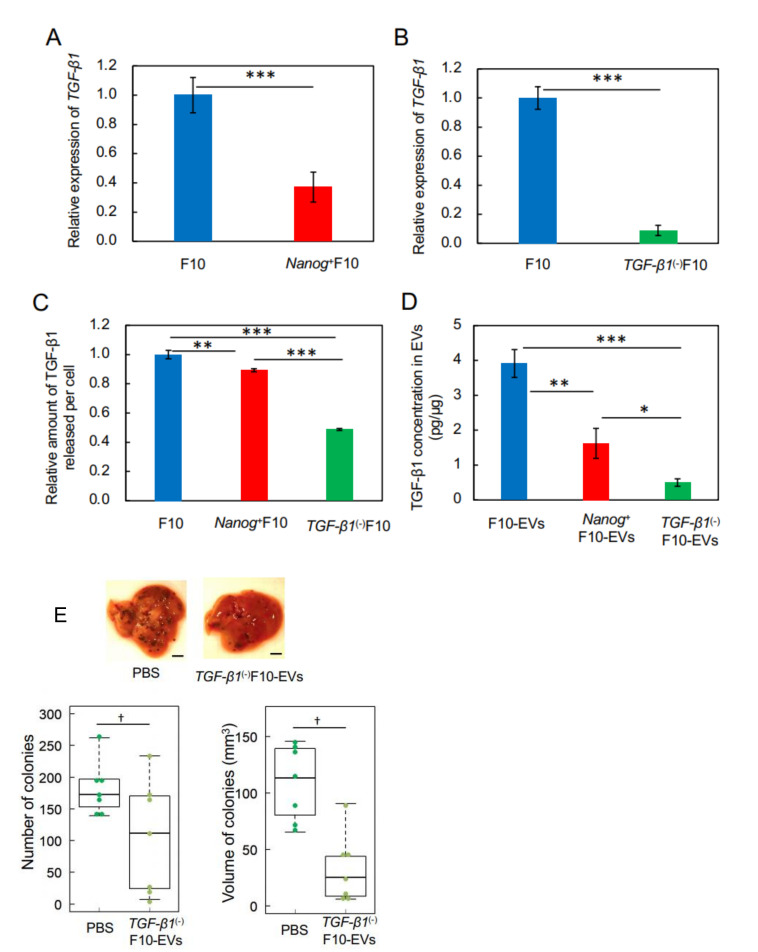
Effects of pre-administration of *TGF-β1*(-)F10-EVs on metastatic potential. (**A**,**B**) Decrease of *TGF-β1* expression in *Nanog*^+^F10 cells and in *TGF-β1*(-)F10 cells, respectively, analyzed by quantitative RT-PCR and shown as the relative values to that in F10 cells. mean ± SD, for *n* = 3. (**C**) The amount of TGF-β1 protein released into the culture medium from *Nanog*^+^F10 cells and *TGF-β1*(-)F10 cells, respectively, analyzed by ELISA and shown as the value per cell that was counted immediately after collecting the culture medium. Results are shown as the relative values to that from F10 cells. mean ± SD, for *n* = 3. (**D**) TGF-β1 concentrations in EVs analyzed by ELISA. mean ± SD, for *n* = 3. (**E**) Effects on the images, number, and volume of metastatic colonies after injection of *TGF-β1*(-)F10 cells. Scale bar, 5 mm; *n* = 7. ***: *p* < 0.001, **: *p* < 0.01, *: *p* < 0.05, †: *p* < 0.1.

In a previous study, we showed that *Nanog* overexpression caused a decrease in the expression of *TGF-β1* in BL6 cells [18]. Here, we likewise found that the level of *TGF-β1* expression in *Nanog*^+^F10 cells decreased by 0.37 fold compared to F10 cells (Figure 3A). Therefore, the effects of this decrease in *TGF-β1* expression on metastasis were investigated using the *TGF-β1* knockdown cell line (*TGF-β1*(-)F10) that was prepared by introducing an shRNA expression vector. The level of *TGF-β1* expression in *TGF-β1*(-)F10 cells was reduced to 0.09 of that in F10 cells (Figure 3B). The amount of TGF-β1 protein released from *Nanog*^+^F10 cells into the culture medium per cell was only 10% smaller than that from F10 cells (Figure 3C); however, release of TGF-β1 protein from *TGF-β1*(-)F10 cells was further reduced to approximately 0.48 fold of that from F10 cells.

With regard to EVs, the amount of TGF-β1 protein in *Nanog*^+^F10-EVs and *TGF-β1*(-)F10-EVs was 0.41 (1.6 pg/μg) and 0.13 (0.5 pg/μg) fold, respectively, of that in F10-EVs (3.9 pg/μg) (Figure 3D). Therefore, *TGF-β1* knockdown had a clear reduction effect on the amount of TGF-β1 protein in *Nanog*^+^F10-EVs. To test the effect of such a low level of TGF-β1 on induced metastasis, we pre-administered *TGF-β1*(-)F10-EVs to mice into the tail vein three times per week for 3 weeks and found that the number and volume of metastatic colonies of *TGF-β1*(-)F10 cells reduced to 0.65 fold and 0.22 fold, respectively, compared to control mice in which PBS was pre-administered (Figure 3E). Therefore, *TGF-β1*(-)F10-EVs showed metastasis-suppressive effects in the same manner as *Nanog*^+^F10-EVs (Figure 2E). These results suggest the concentration-dependent role of TGF-β1 in EVs; a metastasis-suppressive role at low concentration and a metastasis-promotive role at high concentration. The threshold concentration of this role switching may be between 1.6 and 3.9 pg/μg.

### 3.4. Involvement of Macrophages in the In Vivo Effects of Nanog^+^F10-EVs

According to an earlier report [23], the increase in TGF-β1 induced tumor-promotive M2-like macrophages. Even though the amount of TGF-β1 decreased in our study, we nevertheless investigated the potential involvement of macrophages in the tumor-suppressive response.

**Figure 4:**
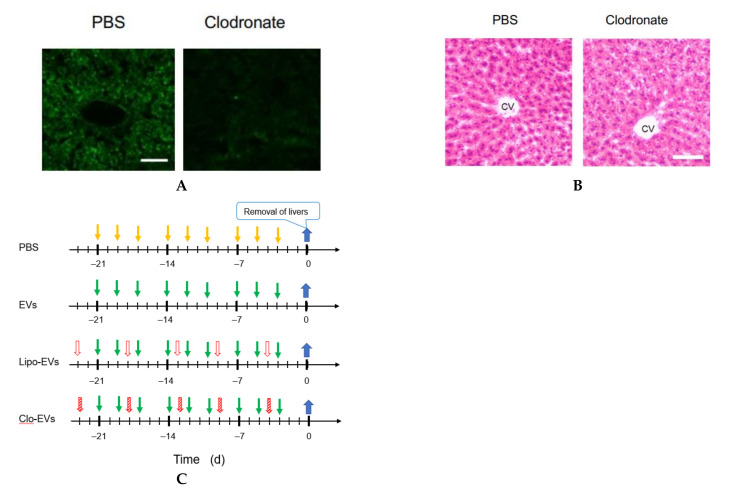

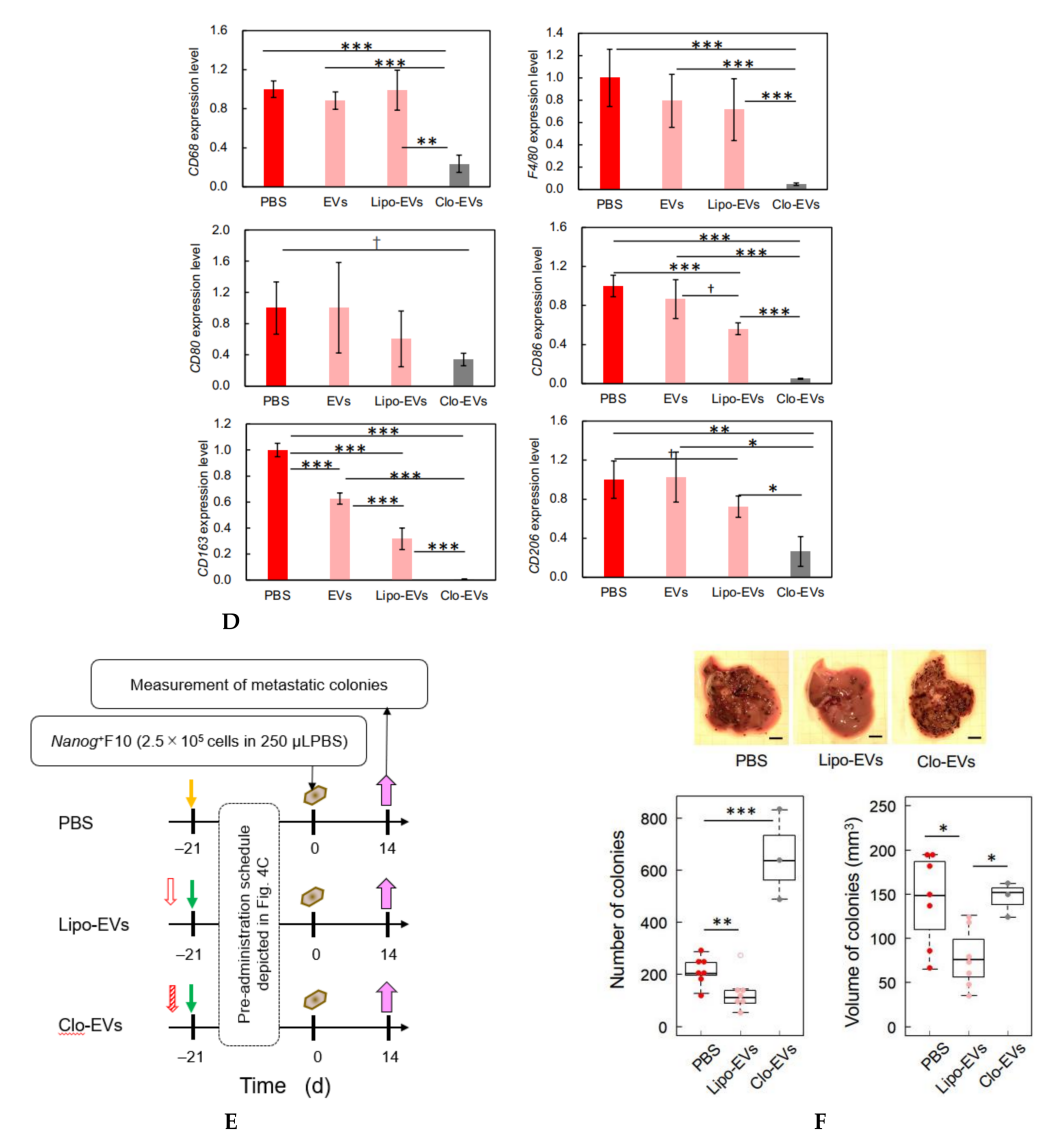
Involvement of macrophages in metastasis suppression by *Nanog*^+^F10-EVs. (**A**) Expression of CD68 in liver tissue slices and its depletion by clodronate treatment. PBS, treated with PBS; Clodronate, treated with clodronate at 12.5 mg/kg mouse body weight; Scale bar, 50 μm. (**B**) Histochemical images of liver tissue sections obtained by HE staining. PBS and Clodronate, same treatment as Figure 4A. CV, central vein; Scale bar, 50 μm.(**C**) Schedule for the pre-administration of *Nanog*^+^F10-EVs with or without clodronate. PBS, control; EVs, *Nanog*^+^F10-EVs; Lipo-EVs, *Nanog*^+^F10-EVs with liposomes containing no clodronate; Clo-EVs, *Nanog*^+^F10-EVs with liposomes containing clodronate. Orange arrow, injection of 100 μL PBS as a control; green arrow, injection of 100 μL PBS containing 5 μg *Nanog*^+^F10-EVs; unfilled red arrow, injection of PBS (in the range of 150–200 μL) containing liposome (62 mg/kg mouse weight) used as a carrier of clodronate; diagonally hatched red arrow, injection of PBS (in the range of 150–200 μL) containing liposome (l62 mg/kg mouse weight) and clodronate (12.5 mg/kg mouse weight); blue arrow, removal of livers. (**D**) Expression of macrophage markers in livers removed on 0 d and analyzed by quantitative RT-PCR. mean ± SD for *n* = 3, *n* = 2 (*CD80* expression for Clo-EVs group). Refer to the legend for Figure 4C about PBS, EVs, Lipo-EVs, and Clo-EVs. (**E**) Schedule for the pre-administration and metastasis experiment. Refer to the legend for Figure 4C about PBS, Lipo-EVs, and Clo-EVs. Brown mark, injection of 250 μL PBS containing 2.5 × 10^5^ cells of *Nanog*^+^F10; violet arrow, removal of livers. (**F**) Effects of clodronate on the metastasis suppression brought about by *Nanog*^+^F10-EVs. Refer to Figure 4E about the schedule of the experiment. Refer to the legend for Figure 4C about PBS, Lipo-EVs, and Clo-EVs. *n* = 7 (PBS, Lipo-EVs), *n* = 3 (Clo-EVs). ***: *p* < 0.001, **: *p* < 0.01, *: *p* < 0.05, †: *p* < 0.1.

In order to deplete macrophages, clodronate-containing liposomes were injected into the tail vein of mice. Five days later, liver tissue sections were prepared and examined by immunostaining of a pan-macrophage marker CD68 to confirm the effect of clodronate. The specific images of macrophages detected in the control (treated with PBS) were lost by the treatment with clodronate (Figure 4A). Under the present condition of clodronate, however, no histochemical abnormality was observed (Figure 4B). Then, according to the schedule depicted in Figure 4C, clodronate-containing liposomes were injected together with *Nanog*^+^F10-EVs into the tail vein of mice for 3 weeks. The livers obtained under 4 conditions (PBS, EVs, Lipo-EVs, and Clo-EVs) were used for quantitative RT-PCR of the following macrophage markers: *CD68* and *F4/80* (pan-macrophage markers), *CD80* and *CD86* (M1-type macrophage markers), and *CD163* and *CD206* (M2-type macrophage markers). Of these markers, only *CD163* showed a significant change between conditions of PBS and EVs (Figure 4D). The expression of *CD163* was reduced to 0.62 of that of the control (PBS). *CD163* expression was completely lost by clodronate-containing liposome (Clo-EVs), indicating that *CD163* decrease was caused by the decrease of macrophages. Therefore, possible effect of *CD163* change on the metastasis, if any, can be disabled by clodronate treatment.

Following pre-administration of *Nanog*^+^F10-EVs, with or without clodronate, *Nanog*^+^F10 cells were injected into the mice at 0 d; 2 weeks later, the number and volume of metastatic colonies were analyzed according to the schedule depicted in Figure 4E. The number and volume of metastatic colonies were reduced by the *Nanog*^+^F10-EVs without clodronate, but this effect of metastasis suppression was lost after co-administration of clodronate (Figure 4F).

### 3.5. Confirmation of the Involvement of Macrophages by In Vitro Tests Using a Macrophage Cell Line J774.1

**Figure 5:**
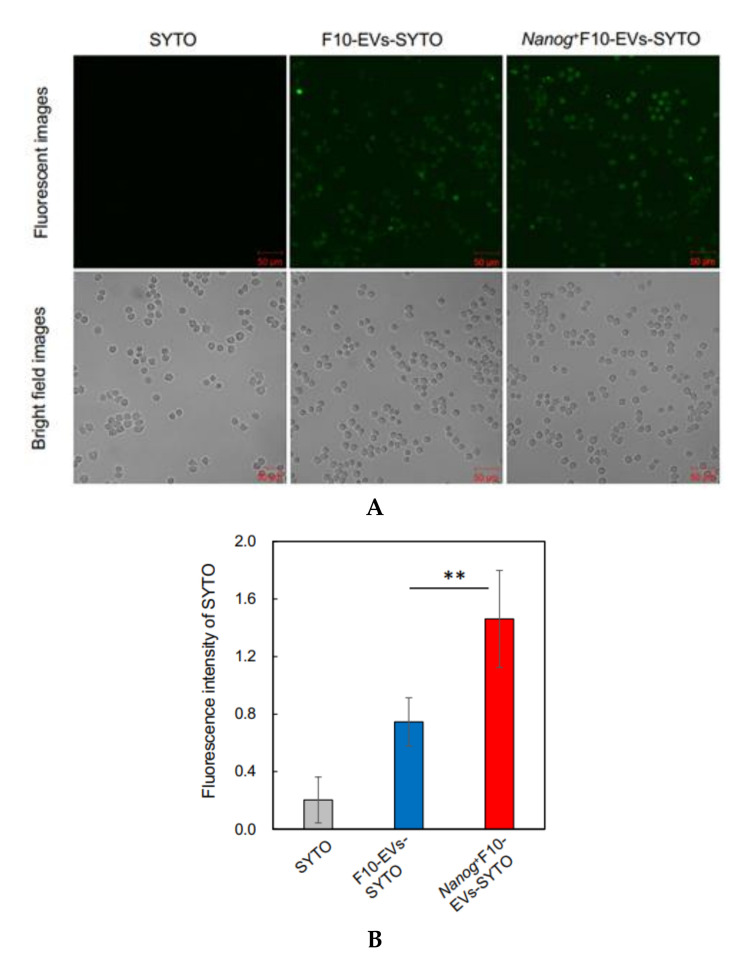

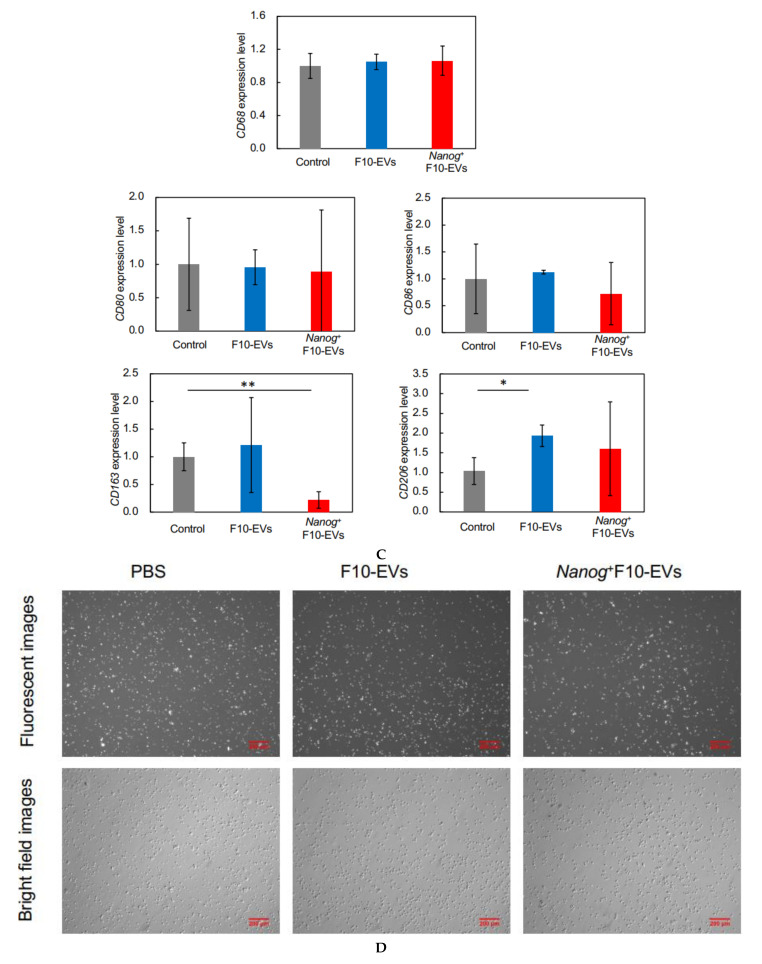

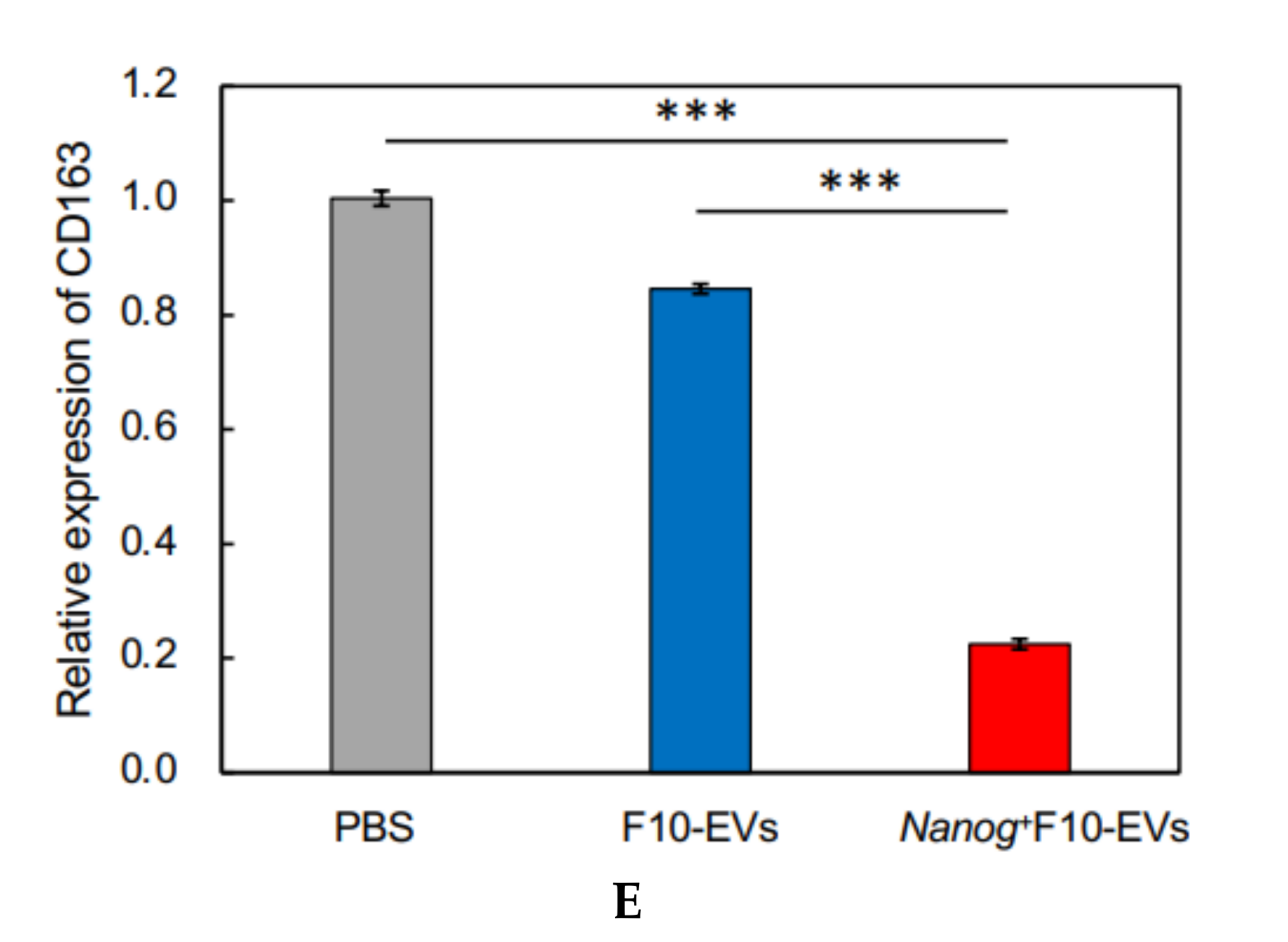
Effects of *Nanog*^+^F10-EVs on the expression of macrophage markers in J774.1 cells. (**A**) Fluorescence microscope images of J774.1 cells that took up SYTO-labelled EVs. J774.1 cells were incubated in medium containing SYTO, F10-EVs-SYTO, and *Nanog*^+^F10-EVs-SYTO, respectively. Scale bar, 50 μm. (**B**) Relative uptake of F10-EVs-SYTO and *Nanog*^+^F10-EVs-SYTO in J774.1 cells. SYTO, control without EVs. Mean ± SD, for *n* = 5. (**C**) Gene expression of macrophage markers analyzed by quantitative RT-PCR. Control, cells incubated in PBS. mean ± SD, for *n* = 3. (**D**) Expression of CD163 protein analyzed by fluorescent immunostaining. J774.1 cells treated with PBS, F10-EVs, and *Nanog*^+^F10-EVs, respectively, and subsequently with an ant-CD163 antibody, and finally with a secondary antibody labelled with AlexaFluor568 (Ex:578 nm, Em: 603 nm). (**E**) Relative number of CD163-positive cells determined by the analysis of fluorescence microscope images of Figure 5D. mean ± SD for *n* = 9 (PBS, *Nanog*^+^F10-EVs), *n* = 8 (F10-EVs). ***: *p* < 0.001, **: *p* < 0.01, *: *p* < 0.05.

F10-EVs and *Nanog*^+^F10-EVs labelled with SYTO, respectively, could be taken up into J774.1 cells (Figure 5A). This result was not due to the non-specific diffusion of SYTO, as free SYTO without EVs was not taken up (Figure 5A SYTO). The analysis of fluorescent images revealed that the uptake of *Nanog*^+^F10-EVs-SYTO was higher than that of F10-EVs-SYTO (Figure 5B).

The expression of macrophage markers in J774.1 cells was analyzed by quantitative RT-PCR. The largest change (decrease) was observed in *CD163* (Figure 5C). Then, the decrease in the CD163 protein was analyzed by fluorescent immunostaining. The cells were reacted with F10-EVs, *Nanog*^+^F10-EVs, or PBS (control). The fluorescent intensities of fluorescent cells were not homogeneous (Figure 5D). The fluorescent intensity and the cell size were analyzed for every fluorescent cell and integrated to determine the fluorescent intensity per unit area. The treatment with *Nanog*^+^F10-EV caused a marked decrease in the fluorescent intensity, indicating the decrease in CD163 (Figure 5E).

## 4. Discussion

**Figure 6:**
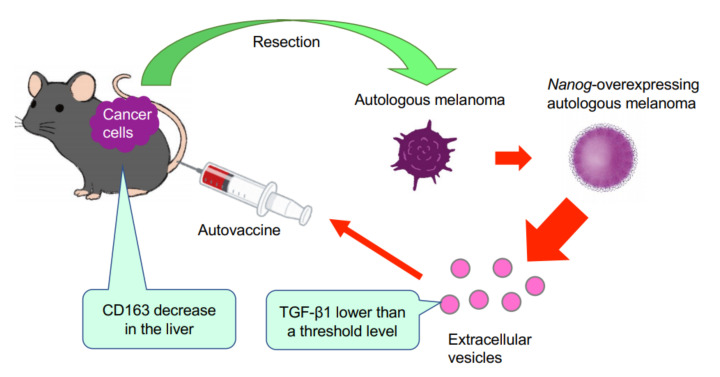
Concept of autovaccine using *Nanog*^+^F10-EVs. F10 cells are resected from primary melanoma. F10 cell line is genetically modified to *Nanog*^+^F10. EVs are separated from *Nanog*^+^F10 cells and administered into the mouse to acquire metastasis-suppressive effects.

This study has demonstrated for the first time that EVs derived from cancer cells with high metastatic potential is an autovaccine candidate based on the concept depicted in Figure 6. An important key factor was *Nanog* overexpression that down-regulated *TGF-β1*. The decrease in TGF-β1 was observed in *Nanog*^+^F10 cells as compared to that in F10 cells. The decrease in TGF-β1 was also observed in EVs derived therefrom. The effects of EVs on the metastasis, however, were exactly opposite for F10-EVs and *Nanog*^+^F10-EVs. The metastasis-suppressive effect obtained with *Nanog*^+^F10-EVs was a surprising finding. Since the concentration-dependent opposite effects were observed, it seemed to be a dual role of TGF-β1.

It has been well discussed that the dual role of TGF-β1 is associated with factors that depend on the conditions of the microenvironment. TGF-β1 is tumor-suppressive in tumors in early stage, but tumor-promotive in advanced cancers. TGF-β1 suppresses ID1 (inhibition of differentiation) in normal cells, but promotes ID1 in cancer cells [19,30]. Activated ID1 promotes metastasis via progression of epitherial–mesenchymal transition (EMT). However, it is unclear what factors are crucial in the difference between microenvironments in normal or early stage tumor cells and in advanced cancer cells. The number of cancer cells and the concentration of TGF-β1 generated thereby might be one of those factors of difference. The concentration-dependent role of TGF-β1 is also suggested from the morphogen like function of TGF-β1 because TGF-β1 is a member of a morphogen superfamily TGF-β [31].

We thought that clearer experimental results should be necessary to claim the concentration-dependent role of TGF-β1. Then, the concentrations of TGF-β1 in F10-EVs and *Nanog*^+^F10-EVs were analyzed and could be determined as 3.9 pg/μg and 1.6 pg/μg, respectively. This suggested the presence of a threshold concentration between 1.6 pg/μg and 3.9 pg/μg; metastasis-suppressive below the threshold and metastasis-promotive above the threshold concentration. Experimental results of TGF-β1 knockdown supported the metastasis-suppressive effect at a concentration lower than the threshold. Although there are few papers that report quantitative studies on the role of TGF-β1 in EVs, we have found a couple of papers that may support the validity of the presence of such a threshold level. Exosomes derived from melanoma A375 cells contained 10–15 pg/μg TGF-β and inactivated T-cells, suggesting a metastasis-promotive role [22]. In contrast, EVs derived from murine colon carcinoma cells that had been genetically modified with overexpression of shRNA for TGF-β1 could induce the tumor growth inhibition [16]. This suggests a metastasis-suppressive effect at a sufficiently low level of TGF-β1.

Since TGF-β1 is involved in immunosuppression via the induction of tumor promotive M2-like macrophages [23], we investigated the responses of six markers selected from three categories of macrophages; pan-macrophage, M1-type, and M2-type macrophage markers. Only CD163, an M2-type macrophage marker, showed a significant decrease in gene expression (Figure 4D) and in protein level (Figure 5C). CD163 positive macrophages are a subpopulation of M2-type macrophages [32]. Proliferation of human sarcoma cells is promoted in co-cultures with CD163-positive macrophages, but not with si-*CD163* [33]. This effect of CD163 is brought about via the production of tumor cell growth factors such as IL6 and CXCL2. IL6 promotes tumor cell proliferation, survival, and metastasis through activation of Stat3 [34,35]. Therefore, the decrease of CD163 is suggestive of a metastasis-suppressive response, and such a response is consistent with the *Nanog*-induced decrease in TGF-β1 in EVs below the threshold level.

Our next study will be directed towards the further fractionation of *Nanog*^+^F10-EVs to purify active components, and the survey of other types of responding immune cells.

## Supplementary Materials

The following supporting information can be downloaded at: https://www.mdpi.com/article/10.3390/curroncol29020088/s1. Figure S1. Full-length gel images for the data of Figure 1A. Figure S2. Examples of metastatic colonies found in Lung and kidney caused by F10 administration. Scale bar: 5 mm. Figure S3. Determination of the volume of metastatic colony. (A) A photograph of liver. A blue triangle indicates a metastatic colony of *Nanog*^+^F10. Bar indicates 5 mm. (B) The shape of each colony was approximated as a spheroid and the following formula was used: *V* = (1/6) π*ab*^2^, where *a* and *b* (=*c*) were the longest and shortest diameters, respectively. Figure S4. Full-length gel images for the data of Figure 2A. Table S1. Primers used for q-PCR.

## References

[1] Kalluri R., LeBleu V.S. (2020). The biology, function, and biomedical applications of exosomes. Science.

[2] Schiera G., Liegro C.M.D., Liegro I.D. (2020). Cell-to-cell communication in learning and memory: From neuro- and glio-transmission to information exchange mediated by extracellular vesicles. Int. J. Mol. Sci..

[3] Sinha D., Roy S., Saha P., Chatterjee N., Bishayee A. (2021). Trends in research on exosomes in cancer progression and anticancer therapy. Cancers.

[4] Psaila B., Lyden D. (2009). The metastatic niche: Adapting the foreign soil. Nat. Rev. Cancer.

[5] Costa-Silva B., Aiello N.M., Ocean A.J., Singh S., Zhang H., Thakur B.K., Becker A., Hoshino A., Mark M.T., Molina H. (2015). Pancreatic cancer exosomes initiate pre-metastatic niche formation in the liver. Nat. Cell Biol..

[6] Saber S.H., Ali H.E.A., Gaballa R., Gaballah M., Ali H.I., Zerfaoui M., Elmageed Z.Y.A. (2020). Exosomes are the driving force in preparing the soil for the metastatic seeds: Lessons from the prostate cancer. Cells.

[7] Yoshida K., Tsuda M., Matsumoto R., Semba S., Wang L., Sugino H., Tanino M., Kondo T., Tanabe K., Tanaka S. (2019). Exosomes containing ErbB2/CRK induce vascular growth in premetastatic niches and promote metastasis of bladder cancer. Cancer Sci..

[8] Ahmadi M., Razaie J. (2020). Tumor cells derived-exosomes as angiogenenic agents: Possible therapeutic implications. J. Transl. Med..

[9] Winkler J., Abisoye-Ogunniyan A., Metcalf K.J., Werb Z. (2020). Concepts of extracellular matrix remodelling in tumour progression and metastasis. Nat. Commun..

[10] Wang J., Veirman K.D., Faict S., Frassanito M.A., Ribatti D., Vacca A., Menu E. (2016). Multiple myeloma exosomes establish a favourable bone marrow microenvironment with enhanced angiogenesis and immunosuppression. J. Pathol..

[11] Czernek L., Düchler M. (2017). Functions of cancer-derived extracellular vesicles in immunosuppression. Arch. Immunol. Ther. Exp..

[12] Plebanek M.P., Angeloni N.L., Vinokour E., Li J., Henkin A., Martinez-Marin D., Filleur S., Bhowmick R., Henkin J., Miller S.D. (2017). Pre-metastatic cancer exosomes induce immune surveillance by patrolling monocytes at the metastatic niche. Nat. Commun..

[13] Kamerkar S., LeBleu V.S., Sugimoto H., Yang S., Ruivo C.F., Melo S.A., Lee J.J., Kalluri R. (2017). Exosomes facilitate therapeutic targeting of oncogenic Kras in pancreatic cancer. Nature.

[14] Fernández-Delgado I., Calzada-Fraile D., Sánchez-Madrid F. (2020). Immune regulation by dendritic cell extracellular vesicles in cancer immunotherapy and vaccines. Cancers.

[15] Rosenberger L., Ezquer M., Lillo-Vera F., Pedraza P.L., Ortúzar M.I., González P.L., Figueroa-Valdés A.I., Cuenca J., Ezquer F., Khoury M. (2019). Stem cell exosomes inhibit angiogenesis and tumor growth of oral squamous cell carcinoma. Sci. Rep..

[16] Rossowska J., Anger N., Wegierek K., Szczygieł A., Mierzejewska J., Milczarek M., Szermer-Olearnik B., Pajtasz-Piasecka E. (2019). Antitumor potential of extracellular vesicles released by genetically modified murine colon carcinoma cells with overexpression of interleukin-12 and shRNA for TGF-β1. Front. Immunol..

[17] Tay B.Q., Wright Q., Ladwa R., Perry C., Leggatt G., Simpson F., Wells J.W., Panizza B.J., Frazer I.H., Cruz J.L.G. (2021). Evolution of cancer vaccines—challenges, achievements, and future directions. Vaccines.

[18] Saito M., Kishi R., Sasai T., Hatakenaka T., Matsuki N., Minagawa S. (2021). Effect of Nanog overexpression on the metastatic potential of a mouse melanoma cell line B16-BL6. Mol. Cell. Biochem..

[19] Seoane J., Gomis R.R. (2017). TGF-β family signaling in tumor suppression and cancer progression. Cold Spring Harb. Perspect. Biol..

[20] Fabregat I., Caballero-Díaz D. (2018). Transforming growth factor-β-induced cell plasticity in liver fibrosis and hepatocarcinogenesis. Front. Oncol..

[21] Suriyamurthy S., Baker D., Dijke P.T., Iyengar P.V. (2019). Epigenetic reprogramming of TGF-β signaling in breast cancer. Cancers.

[22] Düchler M., Czernek L., Peczek L., Cypryk W., Sztiller-Sikorska M., Czyz M. (2019). Melanoma-derived extracellular vesicles bear the potential for the induction of antigen-specific tolerance. Cells.

[23] Zhang F., Wang H., Wang X., Jiang G., Liu H., Zhang G., Wang H., Fang R., Bu X., Cai S. (2016). TGF-β induces M2-like macrophage polarization via SNAIL-mediated suppression of a pro-inflammatory phenotype. Oncotarget.

[24] Percie du Sert N., Hurst V., Ahluwalia A., Alam S., Avey M.T., Baker M., Browne W.J., Clark A., Cuthill I.C., Dirnagl U. (2020). The ARRIVE guidelines 2.0: Updated guidelines for reporting animal research. PLoS Biol..

[25] Peinado H., Alečković M., Lavotshkin S., Matei I., Costa-Silva B., Moreno-Bueno G., Hergueta-Redondo M., Williams C., García-Santos G., Nitadori-Hoshino A. (2012). Melanoma exosomes educate bone marrow progenitor cells toward a pro-metastatic phenotype through MET. Nat. Med..

[26] Wu Y., Deng W., McGinley E.C., Klinke D.J. (2017). Melanoma exosomes deliver a complex biological payload that upregulates PTPN11 to suppress T lymphocyte function. Pigment Cell Melanoma Res..

[27] Li P., Kaslan M., Lee S.H., Yao J., Gao Z. (2017). Progress in exosome isolation techniques. Theranostics.

[28] Dutta S., Reamtong O., Panvongsa W., Kitdumrongthum S., Janpipatkul K., Sangvanich P., Piyachaturawat P., Arthit Chairoungdua A. (2015). Proteomics profiling of cholangiocarcinoma exosomes: A potential role of oncogenic protein transferring in cancer progression. Biochim. Biophys. Acta.

[29] Yan Z., Dutta S., Liu Z., Yu X., Mesgarzadeh N., Ji F., Bitan G., Xie Y.-H. (2019). A Label-Free Platform for Identification of Exosomes from Different Sources. ACS Sens..

[30] Massagué J. (2008). TGFβ in cancer. Cell.

[31] Loomans H.A., Andl C.D. (2015). Intertwining of activin A and TGFβ signaling: Dual roles in cancer progression and cancer cell invasion. Cancers.

[32] Barros M.H., Hauck F., Dreyer J.H., Kempkes B., Niedobitek G. (2013). Macrophage polarisation: An immunohistochemical approach for identifying M1 and M2 macrophages. PLoS ONE.

[33] Shiraishi D., Fujiwara Y., Horlad H., Saito Y., Iriki T., Tsuboki J., Cheng P., Nakagata N., Mizuta H., Bekki H. (2018). CD163 is required for protumoral activation of macrophages in human and murine sarcoma. Cancer Res..

[34] Fisher D.T., Appenheimer M.M., Evans S.S. (2014). The two faces of IL-6 in the tumor microenvironment. Semin. Immunol..

[35] Chonov D.C., Ignatova M.M.K., Ananiev J.R., Gulubova M.V. (2019). IL-6 activities in the tumour microenvironment. part 1. Open Access Maced. J. Med. Sci..

