# Supplementary material for: Anti-Metastatic Function of Extracellular Vesicles Derived from Nanog-Overexpressing Melanoma
Source: Curr Oncol. 2022 Feb 11;29(2):1029–46. doi: 10.3390/curroncol29020088 (PMC8870779; doi:10.3390/curroncol29020088)
Supplement: Supplementary file 1 [file curroncol-29-00088-s001.zip › curroncol-1504984-supplementary.pdf]

Current Oncology

Article

Anti-metastatic function of extracellular vesicles derived from Nanog-overexpressing melanoma

Tomohiro Hatakenaka[1], Nahoko Matsuki[1], Seiya Minagawa[2], Celine Swee May Khoo[1] and Mikako Saito[1,3]*

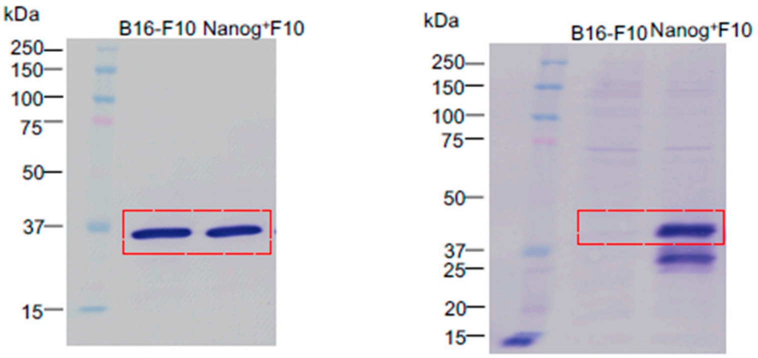

Figure S1. Full-length gel images for the data of Fig. 1A.

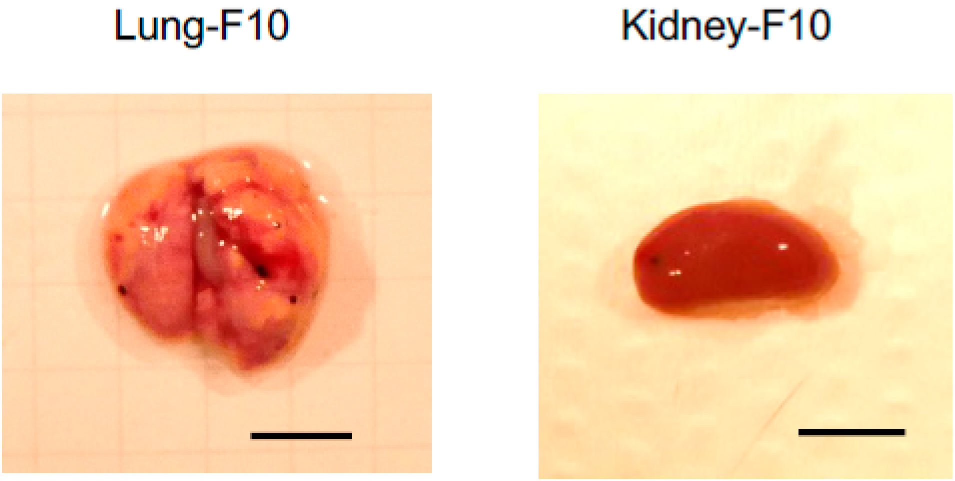

Figure S2. Examples of metastatic colonies found in Lung and kidney caused by F10 administration. Scale bar: 5 mm.

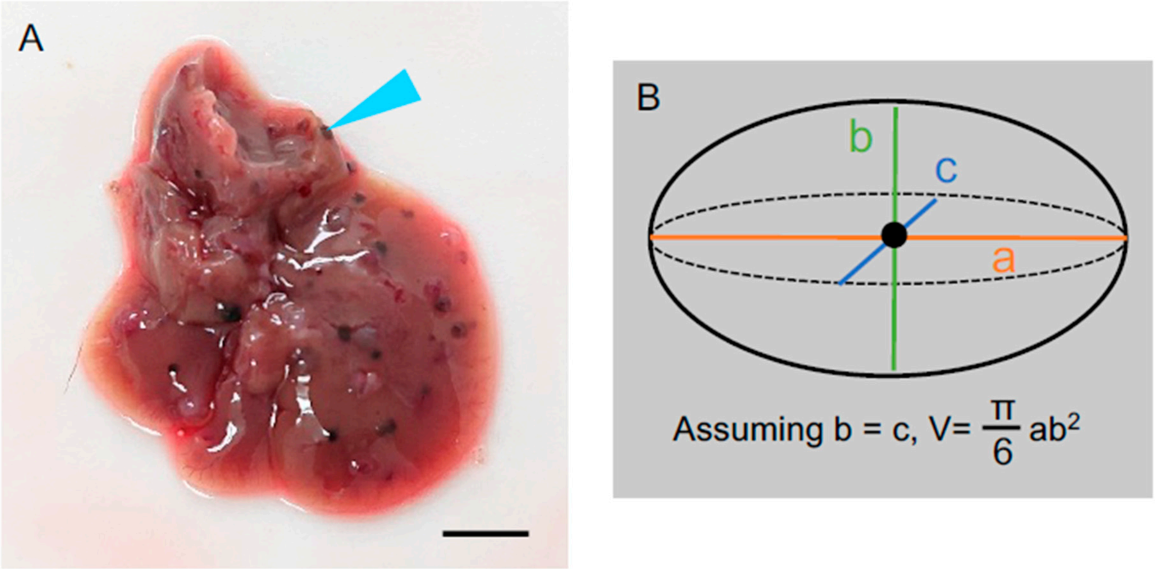

Figure S3. Figure S3. Determination of the volume of metastatic colony. (A) A photograph of liver. A blue triangle indicates a metastatic colony of Nanog+F10. Bar indicates 5 mm. (B) The shape of each colony was approximated as a spheroid and the following formula was used: V = (1/6) πab2, where a and b (= c) were the longest and shortest diameters, respectively.

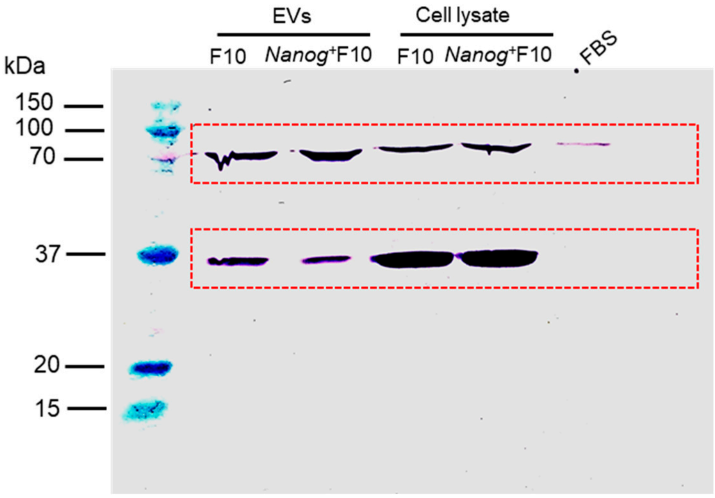

Figure S4. Full-length gel images for the data of Fig. 2A.

Table S1. Primers used for q-PCR.

Genes	Forward primers (5'→3')	Reverse primers (5'→3')
Nanog	ATGCCTGCAGTTTTTCATCC	GAGGCAGGTCTTCAGAGGAA
EGFP	GGGCACAAGCTGGAGTACAAC	CACCTTGATGCCGTTCTTCTG
puro	TCACCGAGCTGCAAGAACTCT	CCCACACCTTGCCGATGT
TGF-β1	ATACCAACTATTGCTTCAGCT CCACAG	GTACTGTGTGTCCAGGCTCCAAATAT
F4/80	CTTTGGCTATGGGCTTCCAGTCC	GCAAGGAGGACAGAGTTTATCGTG
CD68	ACTTCGGGCCATGTTTCTCT	GCTGGTAGGTTGATTGTCGT
CD80	CTGGGAAAAACCCCCAGAAG	TGACAACGATGACGACGACTG
CD86	CATGGGCTTGGCAATCCTTA	AAATGGGCACGGCAGATATG
CD163	GGCTAGACGAAGTCATCTGCAC	CTTCGTTGGTCAGCCTCAGAGA
CD206	ATGCCAAGTGGGAAAATCTG	TGTAGCAGTGGCCTGCATAG
